# Contact Index–Guided Pulsed Field Ablation: A Two‐Phase Validation of the Impedance Change Threshold to Prevent Acute Pulmonary Vein Gaps

**DOI:** 10.1002/joa3.70333

**Published:** 2026-04-04

**Authors:** Ryuta Watanabe, Koichi Nagashima, Hikaru Masuda, Akinori Matsushima, Shu Hirata, Yuji Saito, Masanaru Sawada, Yasuo Okumura

**Affiliations:** ^1^ Division of Cardiology, Department of Medicine Nihon University School of Medicine Tokyo Japan

**Keywords:** atrial fibrillation, Contact Index, impedance, pentaspline catheter, pulmonary vein gaps, pulsed field ablation

## Abstract

**Background:**

Pulsed field ablation (PFA) for atrial fibrillation (AF) requires adequate catheter–tissue contact. The Contact Index visualizes impedance change for the pentaspline catheter, but a validated contact threshold is unknown.

**Objective:**

The objective of this study is to define an impedance‐change cutoff that predicts sufficient contact to prevent acute pulmonary vein gaps (PVG) during pulmonary vein isolation (PVI), and to validate Contact–Index‐guided PVI.

**Methods:**

A prospective, single‐center, two‐phase study was performed. Phase 1 (*n* = 10) measured impedance change (%) at eight antral segments per vein immediately before PFA in basket and flower configurations; the larger per‐segment value was analyzed. Post‐PVI high‐density voltage mapping (PVG ≥ 0.5 mV) was the reference; a receiver operating characteristic analysis identified the cutoff. Phase 2 (*n* = 26) delivered energy only when all target splines met/exceeded the cutoff value obtained from Phase 1.

**Results:**

In Phase 1, PVG occurred in 13/320 segments across 2/10 patients (4/40 veins). Impedance change was lower at PVG‐positive than PVG‐negative segments (median 2 [0–6.5]% vs. 24 [17–32]%, *p* < 0.001). The cutoff value obtained from Phase 1 was ≥ 10% (area under the curve 0.99; sensitivity 100%; specificity 92.1%). In Phase 2, enforcing ≥ 10% yielded no patient‐level PVG (0/26 vs. 2/10; *p* = 0.07) and fewer gaps per vein (0/104 vs. 4/40; *p* = 0.005) and per segment (0/832 vs. 13/320; *p* < 0.001) compared with Phase 1. Procedure time, PVI time, fluoroscopy time, and total applications were similar between phases.

**Conclusions:**

An impedance‐change threshold of ≥ 10% on the Contact Index reliably indicates adequate contact with the pentaspline catheter and, when enforced, reduces acute PVG without prolonging procedures.

## Graphical abstract

This two‐phase study demonstrates that a Contact Index impedance change of ≥ 10% reliably indicates adequate catheter contact during pulsed field ablation (PFA). Enforcing this threshold significantly reduced acute pulmonary vein gaps (PVGs) without prolonging procedure or fluoroscopy times.

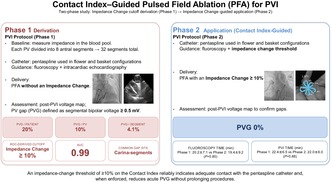

**Abbreviations**
AF: Atrial fibrillation
LA: Left atrium
LI: Left inferior
LS: Left superior
PFA: Pulsed field ablation
PVG: Pulmonary vein gaps
PVI: Pulmonary vein isolation
RI: Right inferior
ROC: Receiver operating characteristic
RS: Right superior
VLCC: Variable‐loop circular catheter

## 1 Introduction

Pulsed field ablation (PFA) has emerged as a novel therapeutic approach for the treatment of atrial fibrillation (AF). Large‐scale clinical trials have demonstrated that PFA shortens procedure time while providing comparable or superior clinical outcomes [1, 2]. Effective PFA delivery depends on adequate catheter–tissue contact. The Tissue Proximity Indication, derived from impedance changes, enables a semi‐quantitative assessment of catheter–tissue contact when using a variable‐loop circular catheter (VLCC, VARIPULSE, Biosense Webster Inc., Irvine, CA) [3, 4, 5, 6]. Recently, the EnSite X mapping system (Abbott, Abbott Park, IL) introduced a new module, the “Contact Index,” which allows real‐time visualization of impedance changes for each electrode on the pentaspline catheter (FARAPULSE, Boston Scientific, Marlborough, MA). However, the optimal impedance change threshold that reliably reflects adequate tissue contact when using the Contact Index has not yet been established. Accordingly, this study using the pentaspline catheter was conducted in two phases. Phase 1 aimed to determine the cutoff value of impedance change for preventing acute pulmonary vein gaps (PVG) after pulmonary vein isolation (PVI), using post‐procedural voltage mapping as the reference. Phase 2 involved clinical validation of PVI guided by the Contact Index, applying the cutoff value determined in Phase 1.

## 2 Methods

### 2.1 Study Design and Population

This was a prospective, single‐center, two‐phase study conducted at Nihon University Itabashi Hospital to evaluate impedance change as a real‐time indicator of catheter–tissue contact during PFA for PVI using a pentaspline catheter. A total of 36 consecutive AF patients who underwent PFA‐based PVI were enrolled between July 2025 and January 2026. Informed consent was obtained through an opt‐out process, and the study protocol was approved by the institutional review board in accordance with the Declaration of Helsinki.

### 2.2 CT‐Based LA Wall Thickness (ADAS)

Between 1 and 7 days before ablation, patients underwent multidetector helical computed tomography (CT) using a 320‐row scanner (Aquilion ONE, Toshiba Medical Systems, Tokyo) [7, 8, 9]. Acquisition parameters were: slice thickness 0.5 mm, gantry rotation 350 ms, tube voltage 120 kV, and tube current 300–580 mA, optimized for high spatial resolution (approximately 0.3 mm). To maintain a heart rate < 65 beats/min, landiolol was administered as needed. A nonionic iodinated contrast agent (Iomeron, Eisai, Tokyo) was injected at 0.07 mL/kg/s for 9 s; bolus‐tracking initiated imaging once contrast reached the left atrium. During end‐expiration, volume acquisition was electrocardiography‐gated (ECG‐gated) to 65%–75% of the R–R interval in sinus rhythm or AF.

“CT data were post‐processed with ADAS 3D (Adas3D Medical SL, Barcelona).” LA wall structural delineation and automated wall‐thickness maps were generated, then manually reviewed and adjusted. The finalized 3D wall‐thickness map was exported and registered to the EnSite X electroanatomic map using standard landmark‐based alignment. For Phase‐1 analyses, wall thickness values were categorized into five categories (0–1, 1–2, 2–3, 3–4, and 4–5 mm) according to ADAS bins [5, 10].

**FIGURE 1:**
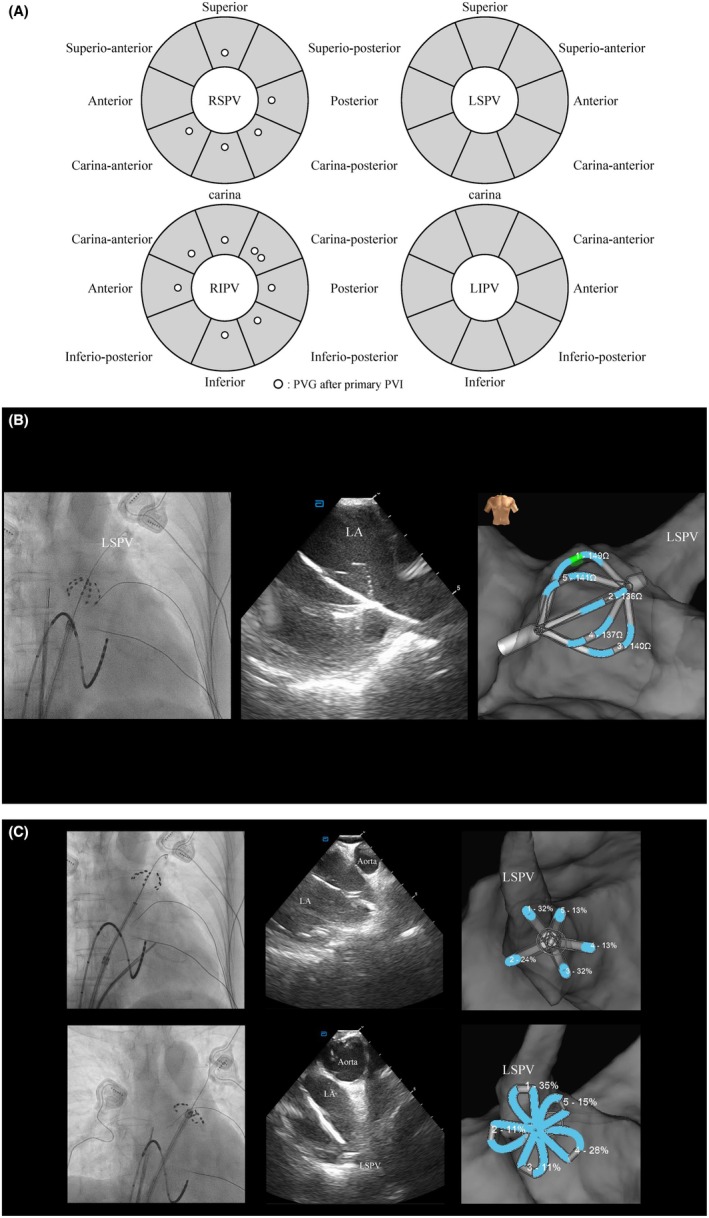
Workflow for measuring impedance change and segment definition. (A) Eight antral segments per pulmonary vein were used for analysis—superior, supero‐anterior, anterior, carina‐anterior, carina, carina‐posterior, posterior, and supero‐posterior for the superior veins; and carina, carina‐anterior, anterior, infero‐anterior, inferior, infero‐posterior, posterior, and carina‐posterior for the inferior veins. For each segment, the larger of the basket‐ or flower‐configuration impedance‐change values was taken as that segment's value. The schematic depicts the eight‐segment framework per vein; segments with acute PVG are marked by circles, with the number of circles indicating how many Phase‐1 cases had PVG at that segment. (B) Baseline impedance acquisition at a LA blood‐pool site (no catheter–tissue contact confirmed by intracardiac echocardiography/fluoroscopy; no local electrograms). (C) Deliberate PV contact with the pentaspline catheter in basket and flower configurations; impedance change (%) was read from electrode #3 on each spline immediately before pulsed‐field delivery.

Each PV segment‐level wall thickness was divided into eight antral segments (32 per patient) as shown in Figure 1A: for the right superior (RS)/left superior (LS) PVs—superior, supero‐anterior, anterior, carina‐anterior, carina, carina‐posterior, posterior, and supero‐posterior; for the right inferior (RI)/left inferior (LI) PVs—carina, carina‐anterior, anterior, infero‐anterior, inferior, infero‐posterior, posterior, and carina‐posterior. Within each segment, wall thickness was sampled around three predefined electrophysiological points from the voltage map, and the segmental value was taken as the modal thickness class based on the ADAS mm bins.

### 2.3 Preparation

PFA‐based PVI was performed under deep sedation (propofol, midazolam, and fentanyl). Mapping and ablation were guided by the EnSite X system. Via femoral venous access, an intracardiac echocardiography catheter was advanced, and a transseptal puncture was performed to introduce a steerable sheath (Faradrive, Boston Scientific) into the left atrium (LA). After transseptal access, the pentaspline catheter was positioned at each PV ostium. Periprocedural anticoagulation consisted of uninterrupted oral anticoagulation, and the activated clotting time was maintained at > 350 s with intravenous heparin.

Before and after PVI, a high‐density LA/PV voltage map was created in sinus rhythm using the HD Grid catheter (Abbott). Bipolar electrograms were recorded with a bandpass filter of 30–500 Hz. If the patient was in AF at the time of mapping, sinus rhythm was restored with internal cardioversion (10–20 J, BeeAT catheter, Japan Lifeline, Tokyo, Japan) prior to mapping. The representative bipolar voltage amplitude for each PV segment (Figure 1A) was retrospectively calculated by averaging the values at the same three sites used for wall‐thickness sampling.

### 2.4 Impedance Change Measurement

Before ablation, a baseline impedance was obtained at a blood‐pool site—with no catheter–tissue contact, confirmed by intracardiac ECG/fluoroscopy and the absence of local electrograms (Figure 1B). Impedance change (%) was defined as the percent change relative to this baseline impedance. Before any ablation at each PV, impedance change was measured on each spline (electrode 3) while the catheter was brought into deliberate PV contact in both basket and flower configurations (Figure 1C). For each segment, impedance change was measured in both configurations, and the largest value was taken as the impedance change for that segment. The eight PV segments (Figure 1A) were then linked to the corresponding ADAS and electrophysiological segments to enable comparisons with PVG and impedance change.

### 2.5 Ablation Protocol

PVI was performed using the pentaspline catheter, a pentaspline over‐the‐wire PFA catheter with four electrodes on each spline [1]. According to published protocols [4], eight PFA applications were delivered per vein—four in the basket configuration and four in the flower configuration—with an approximately 36° rotation between pairs. Each application consisted of five consecutive pulse trains totaling 2.5 s. In Phase 1, PF energy delivery was guided by the intracardiac echocardiography, fluoroscopy, and local electrograms, whereas in Phase 2 the energy delivery was guided by fluoroscopy and local electrograms under the Contact Index guidance without intracardiac echocardiography. After each PVI, a high‐density voltage map was created to confirm acute residual PVGs, defined as residual voltage ≥ 0.5 mV. Additional applications were delivered when PVGs were detected, and the procedural endpoint was complete electrical isolation of all PVs.

### 2.6 Phase 1: Derivation Phase

The Phase 1 included 10 patients (mean age 59.1 ± 14.6 years; 9 men). During this phase, impedance change values were blinded to the operators. Impedance change (%) was recorded for each spline of the pentaspline catheter immediately before each PFA application in both catheter configurations (basket and flower). After PVI, a high‐density voltage map was created to identify acute residual PVGs. Retrospectively, the optimal cutoff value of impedance change for preventing acute PVGs was determined.

### 2.7 Phase 2: Validation Phase

The Phase 2 included 26 patients (mean age 66.1 ± 12.0 years; 15 men). In this phase, operators did not use intracardiac echocardiography during PF energy delivery and performed PVI under the Contact Index guidance, using the cutoff value derived from Phase 1. Energy delivery proceeded only when all target splines met or exceeded the impedance threshold; otherwise, the catheter was repositioned. Operators avoided applying excessive mechanical pressure to artificially increase impedance readings.

### 2.8 Endpoints

In both phases, the primary endpoint was the absence of acute PVGs on post‐PVI voltage mapping. Secondary endpoints included total procedure time, fluoroscopy time, and the number of applications required for complete isolation.

### 2.9 Statistical Analysis

Continuous variables are expressed as mean ± SD or median [interquartile range], as appropriate, and were compared using the Student's *t*‐test or Mann–Whitney *U* test. Categorical variables were compared using the chi‐square or Fisher's exact test, as appropriate. When more than two groups were compared, the Kruskal–Wallis test was used.

In Phase 1 (derivation), receiver operating characteristic (ROC) curves were constructed at the per‐segment level to evaluate impedance change against the mapping‐based PVG reference (residual voltage ≥ 0.5 mV). The area under the curve was calculated, and the optimal cutoff was determined by the Youden index with corresponding sensitivity and specificity. Additionally, per‐segment logistic regression of PVG on ordered 1‐mm LA wall thickness categories estimated odds ratios with 95% confidence intervals.

In Phase 2 (validation), the primary endpoint was the absence of PVG on the post‐PVI voltage map, analyzed at three granularities (per‐patient, per‐vein, per‐segment).

When any expected count was < 5, the Fisher's exact test was applied.

Secondary continuous variables (procedure time, fluoroscopy time, and number of applications per vein) were compared using the same methods as describedearlier.

All analyses were performed using JMP 18.2.2 (SAS Institute, Cary, NC, USA).

## 3 Results

### 3.1 Phase 1: Derivation Phase

#### 3.1.1 Patient and Procedural Characteristics

Baseline characteristics of Phase 1 are shown in Table 1. The mean CHADS_2_ score was 1.4 ± 0.8. Acute PVG occurred in 2 of 10 patients (20%).

**TABLE 1:** Patient and procedural characteristics of the phase 1.

	Phase 1, *n* = 10	PVG, *n* = 2	Non‐PVG, *n* = 8	*P* value
**Clinical characteristics**				
Age, years	59.1 ± 14.6	55.6 ± 13.8	73.0 ± 9.9	0.14
Male sex	9 (90%)	1 (50%)	8 (100%)	0.20
Paroxysmal AF	5 (50%)	2 (100%)	3 (38%)	0.44
Body mass index, kg/m^2^	26.4 ± 1.5	27.8 ± 2.0	26.1 ± 1.3	0.15
Heart failure	1 (10%)	0 (0%)	1 (13%)	1.00
Hypertension	8 (80%)	2 (100%)	6 (75%)	1.00
Diabetes mellitus	4 (40%)	0 (0%)	4 (50%)	0.47
History of stroke	0 (0%)	0 (0%)	0 (0%)	1.00
CHADS_2_ score	1.4 ± 0.8	1.5 ± 0.7	1.4 ± 0.9	0.86
Echocardiographic variables				
Left ventricular ejection fraction, %	69.1 ± 6.2	76.1 ± 10.5	67.4 ± 4.1	0.076
Left atrial volume, mL	54.0 ± 15.0	60.7 ± 0.0	52.4 ± 17.0	0.53
E/e'	10.8 ± 4.0	11.7 ± 2.1	8.4 ± 2.3	0.11
Cardiac biomarkers				
N‐terminal pro‐brain natriuretic peptide, pg/mL	246 [92–437]	419 [289–548]	330 [86–949]	0.36
Hemoglobin A1c, %	6.3 ± 0.8	6.0 ± 0.4	6.3 ± 0.8	0.55
**Ablation related characteristics**				
The total number of applications for complete PVI	44.0 ± 5.4	52.0 ± 2.8	42.0 ± 3.7	0.008
The number of applications for primary PVI	41.4 ± 3.5	39.0 ± 2.4	42.0 ± 3.7	0.31
LSPV	12.4 ± 2.1	11.0 ± 1.4	12.8 ± 2.1	0.31
LIPV	8.2 ± 0.6	8.0 ± 0	8.3 ± 0.7	0.65
RSPV	11.8 ± 1.8	12.0 ± 0	11.8 ± 2.0	0.87
RIPV	9.0 ± 1.7	8.0 ± 0	9.3 ± 1.8	0.38
The number of additional applications		13.0 ± 4.2	0	
Procedural time				
PVI duration time (min)	22.4 ± 6.5	29.0 ± 12.7	20.8 ± 3.9	0.11
Fluoroscopic time (min)	20.2 ± 7.1	20.9 ± 7.2	20.0 ± 7.6	0.88
Total procedural time (min)	88.2 ± 21.6	103.5 ± 9.2	84.4 ± 22.5	0.29

*Note:* Values are presented as mean ± SD, median [interquartile range], or *n* (%).

*Abbreviations:* AF, Atrial fibrillation; LI, Left inferior; LS, Left superior; PVI, Pulmonary vein isolation; RI, Right inferior; RS, Right superior.

Patient characteristics were generally comparable between groups, except for a trend toward higher left ventricular ejection fraction in patients with PVG (76.1% ± 10.5% vs. 67.4% ± 4.1%, *p* = 0.076).

Procedural metrics, including total PVI, fluoroscopy, and procedure times, did not differ significantly (Table 1). However, patients with PVG required more total applications to achieve complete isolation (52.0 ± 2.8 vs. 42.0 ± 3.7, *p* = 0.008).

#### 3.1.2 Predictors of Acute PVG

**FIGURE 2:**
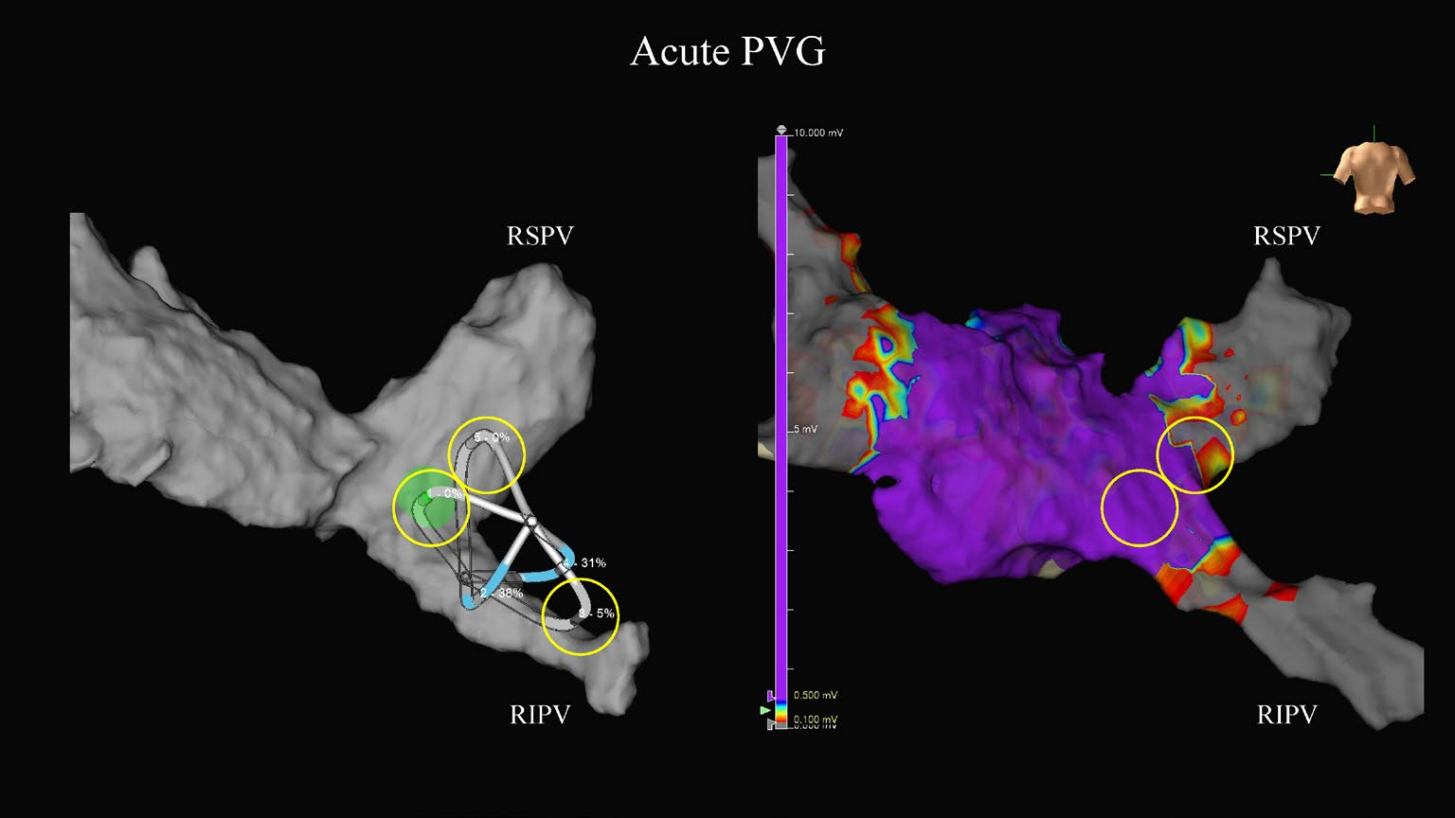
Representative case with acute PVG on post‐PVI voltage map. Post‐procedural high‐density voltage mapping demonstrates residual antral electrograms indicating acute PVG after first‐pass applications; gaps are annotated on the right‐sided veins (RSPV/RIPV) corresponding to segments shown in Figure 1C.

Acute PVG was identified in 13 segments from 2 patients, affecting 2 RSPVs and 2 RIPVs (segmental distribution, Figure 1A; representative case, Figure 2).

At a blood‐pool (non‐contact) site, the baseline impedance was 182 [168–189] Ω and showed a positive correlation with hematocrit (*r* = 0.71, *p* = 0.021). Impedance change was substantially lower at PVG‐positive than PVG‐negative segments (2 [0–6.5]% vs. 24 [17–32]%, *p* < 0.001). Consistently, the calculated impedance at PVG‐positive segments was lower than at PVG‐negative segments (182 [150–189] Ω vs. 224 [203–245] Ω, *p* < 0.001).

Among PVG‐negative veins, median impedance change differed by PV (*p* < 0.001), but not between basket and flower configurations (*p* = 0.082: Figure S1). Among PVG‐positive veins, the median impedance change was 1.0 [0.5–4.5]% for the RSPV and 4.0 [0–8.5]% for the RIPV, both markedly lower than in PVG‐negative counterparts (RSPV, 30.5 [21.3–36.8]%; RIPV, 30.5 [21.3–36.8]%; both *p* < 0.001). In the PVG‐positive subset, impedance change was also significantly lower in both catheter configurations—3.0 [0–6.8]% in the basket and 1.0 [0.5–7.5]% in the flower—compared with PVG‐negative configurations (basket, 25.0 [17.8–34.0]%; flower, 22.0 [16–31]%; both *p* < 0.001; Figure S1).

**FIGURE 3:**
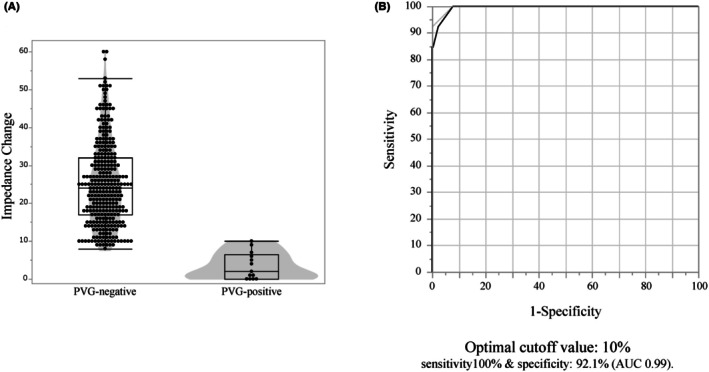
Impedance change by PVG status and ROC analysis. (A) Distribution of impedance change (%) at PVG‐negative versus PVG‐positive segments (points with box/violin overlays). (B) ROC analysis for predicting PVG absence from impedance change: Area under the curve 0.99; sensitivity 100% and specificity 92.1% at the optimal cutoff of 10%.

The distribution of impedance change by PVG status is shown in Figure 3A; the dashed line marks the ROC‐derived cutoff of 10%, and ROC analysis confirmed 10% as optimal (area under the curve 0.99; sensitivity 100%; specificity 92.1%, Figure 3B).

LA bipolar voltage did not differ between PVG‐positive and PVG‐negative segments, but LA wall thickness (ADAS) showed a graded association with PVG across 0–1, 1–2, 2–3, 3–4, and 4–5 mm categories: PVG prevalence increased with thicker categories (Cochran–Armitage Z = 2.17, *p* = 0.030). In an ordinal logistic model (1–5 score), each one‐category increase in thickness was associated with higher odds of PVG (odds ratio 2.01; 95% confidence intervals 1.06–3.78; *p* = 0.032). A stratified analysis of impedance change across the five LA wall thickness categories revealed a statistically significant difference (the Kruskal–Wallis *p* = 0.007), with lower impedance changes observed in thicker segments. The median impedance change was 25 [16–35]% for 0–1 mm, 25 [17–35]% for 1–2 mm, 21 [13–27]% for 2–3 mm, 14 [10–27]% for 3–4 mm, and 20 [16.3–27.5]% for 4–5 mm. When further stratified by PVG status within each thickness category, impedance change was lower in PVG‐positive than PVG‐negative segments (1–2 mm [PVG‐positive, *n* = 4]: 5.5 [1–9.3]% vs. 25 [18–36]%; 2–3 mm [PVG‐positive, *n* = 7]: 2 [0–6]% vs. 22 [15–27]%; 3–4 mm [PVG‐positive, *n* = 2]: 0.5 [0–1]% vs. 22 [11–29]%). No PVG‐positive segments were observed in the 4–5 mm category.

### 3.2 Phase 2

Baseline characteristics (Table 2) were similar between phases except for higher body mass index and diabetes prevalence in Phase 1.

**TABLE 2:** Clinical and ablation related characteristics of the phase 1 and Phase 2 patients.

	Phase 1 (*n* = 10)	Phase 2 (*n* = 26)	*P* value
**Clinical characteristics**			
Age, years	59.1 ± 14.6	66.1 ± 12.0	0.15
Male sex	9 (90%)	15 (58%)	0.11
Paroxysmal AF	5 (50%)	15 (58%)	0.68
Body mass index, kg/m^2^	26.4 ± 1.5	24.7 ± 3.5	0.15
Heart failure	1 (10%)	4 (15%)	1.00
Hypertension	8 (80%)	15 (58%)	0.27
Diabetes mellitus	4 (40%)	2 (8%)	0.039
History of stroke	0 (0%)	0 (0%)	1.00
CHADS_2_ score	1.4 ± 0.8	1.0 ± 0.8	0.19
Echocardiographic variables			
Left ventricular ejection fraction, %	69.1 ± 6.2	68.9 ± 6.8	0.93
Left atrial volume, mL	54 ± 15	52 ± 17	0.78
E/e'	9.1 ± 2.5	10.9 ± 4.8	0.26
Cardiac biomarkers			
N‐terminal pro‐brain natriuretic peptide, pg/mL	246 [92–437]	281 [94–578]	0.74
Hemoglobin A1c, %	6.3 ± 0.8	5.8 ± 0.7	0.06
**Ablation related characteristics**			
The total number of applications for complete PVI	44.0 ± 5.4	45.0 ± 4.7	0.59
The number of applications for primary PVI	41.4 ± 3.5	45.0 ± 4.7	0.035
LSPV	12.4 ± 2.1	12.9 ± 2.3	0.51
LIPV	8.2 ± 0.6	9.2 ± 1.8	0.009
RSPV	11.8 ± 1.8	12.7 ± 2.5	0.34
RIPV	9.0 ± 1.7	10.1 ± 2.2	0.14
Additional ablation strategy			
LA posterior wall isolation (PFA)	1 (10%)	6 (23%)	0.66
Superior vena cava isolation (PFA)	2 (20%)	1 (4%)	0.18
Mitral isthmus (radiofrequency ablation)	0 (0%)	1 (4%)	1.00
Cavotricuspid isthmus (radiofrequency ablation)	3 (30%)	10 (38%)	0.72
Procedural time			
PVI duration time (min)	22.4 ± 6.5	22.0 ± 6.0	0.88
Fluoroscopic time (min)	20.2 ± 7.1	19.4 ± 9.2	0.80
Total procedural time (min)	88.2 ± 21.6	103.6 ± 41.5	0.28
The Incidence of PVG			
PVs with PVG	4 (10%)	0 (0%)	0.005
Segments with PVG	13 (4.1%)	0 (0%)	< 0.001

*Note:* Values are presented as mean ± SD, median [interquartile range], or *n* (%).

*Abbreviations:* AF, Atrial fibrillation; LI, Left inferior; LS, Left superior; PFA, Pulsed field ablation; PVG, Pulmonary vein gaps; PVI, Pulmonary vein isolation; RI, Right inferior; RS, Right superior.

Using the Phase 1–derived cutoff (impedance change ≥ 10%), no PVGs occurred in Phase 2 (0/26 patients vs. 2/10 in Phase 1, *p* = 0.07; 0/104 vs. 4/40 veins, *p* = 0.005; 0/832 vs. 13/320 segments, *p* < 0.001).

The number of primary PVI applications was slightly greater in Phase 2 (45.0 ± 4.7 vs. 41.4 ± 3.5, *p* = 0.035), particularly at the LIPV (*p* = 0.009), but the total number of applications (45.0 ± 4.7 vs 44.0 ± 5.4, *p* = 0.59), PVI time (22.0 ± 6.0 min vs. 22.4 ± 6.5 min, *p* = 0.88), fluoroscopy time (19.4 ± 9.2 min vs. 20.2 ± 7.1 min, *p* = 0.80), and procedure time (103.6 ± 41.5 min vs. 88.2 ± 21.6 min, *p* = 0.28) were comparable (Table 2).

Additional ablation beyond PVI was required in three Phase 2 patients and two Phase 1 patients.

### 3.3 Complications

No procedure‐related complications occurred in either phase.

## 4 Discussion

### 4.1 Main Findings

First, we characterized the impedance‐based contact distribution during applications across the 4PVs and its association with PVG, and identified an optimal criterion for the pentaspline PFA catheter: an impedance change cutoff of 10% derived from ROC analysis (area under the curve 0.99) that discriminated segments with and without acute PVG. Second, prospective application of this impedance change‐guided strategy in Phase 2 was associated with the absence of PVG at the patient level and with significantly lower PVG at the per‐vein and per‐segment levels. Procedural efficiency was maintained, with comparable total applications and PVI times between phases. Third, ADAS‐based LA wall thickness showed a positive association with PVG in Phase 1, suggesting substrate‐level contributors in addition to contact quality.

### 4.2 Impedance as a Contact Indicator

Using the Contact Index, this study quantified impedance change (%) at electrode 3 on each spline with a nearby non‐contact baseline, and validated the metric against post voltage mapping. Segments with PVG showed lower impedance change than segments without PVG (median 2% vs. 24%), supporting impedance change as a practical surrogate of tissue contact for the pentaspline system.

Absolute impedance is influenced by patient hematocrit and myocardial location [11, 12], making it unsuitable as a contact marker. Consistent with this, in this study cohort the patient‐level mean non‐contact baseline impedance correlated with hematocrit. A relative, baseline‐referenced impedance change within each patient provides a more robust and individualized index. Prior reports with a VLCC catheter used a tissue proximity indication around 7% to denote contact [5, 6]. The optimal cutoff (10%) in this study, differs numerically, plausibly reflecting the dependence of measured impedance on electrode size and materials [13]. Nevertheless, the core principle remains: catheter–tissue contact is indexed by a reproducible percent rise from a blood‐pool baseline. Therefore, the ≥ 10% threshold identified in this study should be considered specific to the combination of the pentaspline catheter and the EnSite X mapping system. While the methodology—guiding ablation based on a relative impedance rise—is likely adaptable to other PFA systems, we acknowledge that multicenter studies across different operators and platforms are necessary to confirm the external validity and optimal cutoffs for other setups. We operationalized this prospectively by requiring impedance change ≥ 10% on all target splines before energy delivery in Phase 2, thereby minimizing contact‐poor “weak links.” Extremely high impedance changes, particularly at distal PV sites [11], may instead indicate excessive tissue engagement. In such cases, a slight pullback toward the antrum helps maintain effective contact while avoiding deep intravein positioning. This balanced approach—avoiding both insufficient and excessive contact—appears key to safe, reproducible impedance‐guided PFA.

### 4.3 Procedural Efficiency

Impedance change guidance increased the number of initial circumferential applications (particularly at the LIPV), but the total applications, PVI time, fluoroscopy time, and overall procedure time were not different between phases. Thus, enforcing impedance change ≥ 10% did not prolong procedures and may front‐load applications to ensure completeness, without net time penalties. From a workflow perspective, real‐time impedance change feedback helped operators avoid delivering energy at subthreshold segments, prompted timely repositioning, and reduced the need for extensive intracardiac echocardiography manipulation to visually verify contact, which likely contributed to similar overall times despite more initial applications. A simple, visible rule (≥ 10%) may therefore streamline decision‐making while safeguarding lesion quality.

### 4.4 Comparative Context: First‐Pass Isolation and Anatomical Distribution

High acute success rates for PFA‐PVI have been reported, but many series provide limited detail on first‐pass isolation or the anatomical distribution of PVG [14, 15]. In a multicenter comparison of pentaspline and fixed‐loop PFA catheters, first‐pass isolation was higher with the pentaspline catheter (79.9%) than with the fixed‐loop device (62.3%; *p* < 0.001), with acute PVG most frequent at LSPV, followed by RSPV, LIPV, and RIPV [16]. For VLCC systems, first‐pass isolation around 81% has been reported [5]. Although direct cross‐platform or cross‐study comparisons are limited, the discrepancy between loop‐type platforms (fixed‐loop 62.3 vs. VLCC 81%), despite broadly similar geometry, suggests that an explicit contact proxy (e.g., tissue proximity indication in VLCC) may enhance first‐pass isolation. Consistent with this, in the cohort first‐pass isolation was 80% without impedance change guidance in Phase 1 and improved to 100% with impedance change‐guided PVI in Phase 2.

Notably, acute PV gaps clustered at the pulmonary vein carina, where ADAS‐derived wall thickness was greatest. Thicker antral segments—especially the carina—appeared more prone to residual gaps when contact was suboptimal, emphasizing the need for assured tissue engagement in these regions. The stratified analysis showed that impedance change values were significantly lower in thicker segments (*p* = 0.007), likely reflecting the anatomical challenge of stabilizing the catheter in these regions. In addition, when impedance change was stratified by PVG status within the same wall thickness categories, PVG‐positive segments consistently exhibited markedly lower impedance change than PVG‐negative segments within the same thickness range. Among PVG‐negative segments, the median impedance change in the thickest wall‐thickness categories remained above 10%. This clarifies that while thicker myocardium makes it harder to generate high impedance signals, the 10% threshold is still achievable and applicable. Although thicker myocardium may pose a greater challenge in achieving complete acute lesion completeness, the Phase 2 results—demonstrating zero acute PVGs on post‐PVI voltage mapping with the ≥ 10% cutoff—suggest that this threshold is sufficient to prevent acute gaps even in thicker carinal regions. Rather than requiring a numerically higher impedance target, these segments likely require more diligent catheter manipulation to strictly satisfy the ≥ 10% criterion, given their complex geometry.

### 4.5 Clinical Implications

A single, quantitative contact rule (impedance change ≥ 10%) is easy to implement and portable across operators. The significant reductions in PVG at the vein and segment levels, together with neutral procedure times, argue for routine impedance change monitoring during pentaspline PVI. Because acute gaps are a mechanistic precursor of late reconnection, impedance change‐guided delivery may improve durability; this hypothesis requires longitudinal confirmation.

Furthermore, these findings highlight the practical integration of anatomical knowledge—with or without pre‐procedural ADAS—and Contact Index guidance. The data demonstrate that anatomically thick regions, such as the carina, are inherently challenging for achieving high impedance changes. When ablating in these vulnerable zones, the clinical strategy should not be to apply a different impedance target or indiscriminately increase the number of applications. Instead, operators should use the Contact Index as a strict real‐time execution check. In these regions, operators must prioritize meticulous catheter manipulation—such as adjusting the axis or alternating between flower and basket configurations—to ensure the ≥ 10% threshold is consistently achieved.

### 4.6 Limitations

First, this was a single‐center study with a modest sample size (Phase 1, *n* = 10; Phase 2, *n* = 26), which limits precision and generalizability. However, the prospective validation in the expanded Phase 2 cohort demonstrated a consistent absence of acute PV gaps, reinforcing the robustness of the derived threshold despite the limited sample size. Second, tissue contact and lesion quality were adjudicated by bipolar voltage mapping (< 0.5 mV) rather than by contact force or histopathology; although clinically relevant, this surrogate may misclassify some segments. Third, the impedance‐change metric was available only from a single sensing electrode (the third electrode), precluding direct assessment of contact at the remaining electrodes. Although this study deliberately selected electrode #3 to maximize spatial specificity at the anatomical apex of the spline rather than using a broader whole‐spline measurement, the contact conditions vary along the spline. The present system lacks the capability to simultaneously and independently monitor impedance changes for all four individual electrodes. Future technological advancements enabling individual electrode‐level assessment may allow for a more comprehensive estimation of overall catheter‐tissue interaction. Finally, the 10% cutoff was derived and optimized in Phase 1. The exceptionally high discriminatory performance (AUC = 0.99) observed in this derivation cohort must be interpreted with caution, as it is likely inflated by the small sample size and the low number of PVG‐positive events (*n* = 13). Furthermore, while the single‐electrode model maximizes spatial specificity, it remains unknown whether assessing impedance change through tripolar techniques or multi‐electrode averaging would yield similar performance. This study hypothesize that averaging signals across the spline might dilute the localized tissue‐contact signal with blood‐pool impedance from non‐contacting electrodes, potentially altering diagnostic accuracy. Therefore, external, multicenter validation across platforms is warranted to confirm the true generalizability of this metric.

### 4.7 Conclusions

In this two‐phase study of pentaspline PFA‐PVI, an impedance change ≥ 10% accurately identified catheter–tissue contact (area under the curve 0.99) and, when applied prospectively, reduced acute PV gaps without prolonging procedure metrics. LA–PV wall thickness correlated with PVG, underscoring substrate effects when contact is suboptimal. Incorporating the ≥ 10% threshold as an adjunct to fluoroscopy and electrogram guidance improves lesion completeness—particularly at carinal segments.

## Ethics Statement

The study involving human participants were performed according to protocols approved by the Institutional Review Board of Nihon University Itabashi Hospital (RK‐240312‐1).

## Consent

Informed consent was obtained through an opt‐out process, in accordance with institutional and ethical guidelines.

## Conflicts of Interest

Yasuo Okumura has received research funding from the Medtronic Japan, MicroPort CRM Japan, Bayer Healthcare, has accepted remuneration from AstraZeneca and Johnson & Johnson, and belongs to the endowed departments of the Boston Scientific Japan, Abbott Medical Japan, Japan Lifeline, Medtronic Japan, and BIOTRONIK Japan. Koichi Nagashima has received speaker honoraria from Johnson & Johnson/Biosense Webster, Medtronic Japan, and Boston Scientific Japan. All other authors declare no conflicts of interest.

## Supporting information


**Data S1:** Figure S1. Distribution of impedance change (%) stratified by pulmonary vein gap (PVG) status.
**Left panel:** By pulmonary vein—left superior (LSPV), left inferior (LIPV), right superior (RSPV), and right inferior (RIPV)—shown separately for PVG‐negative and PVG‐positive segments.
**Right panel:** By FARAPULSE catheter deployment configuration (basket vs. flower), again split by PVG status. Overall, PVG‐positive segments show markedly lower impedance change than PVG‐negative segments across veins and configurations.

## Data Availability

Research data are not shared.
