# Supplementary figures and images for: Contact Index–Guided Pulsed Field Ablation: A Two‐Phase Validation of the Impedance Change Threshold to Prevent Acute Pulmonary Vein Gaps
Source: J Arrhythm. 2026 Apr 4;42(2):e70333. doi: 10.1002/joa3.70333 (PMC13051895; doi:10.1002/joa3.70333)

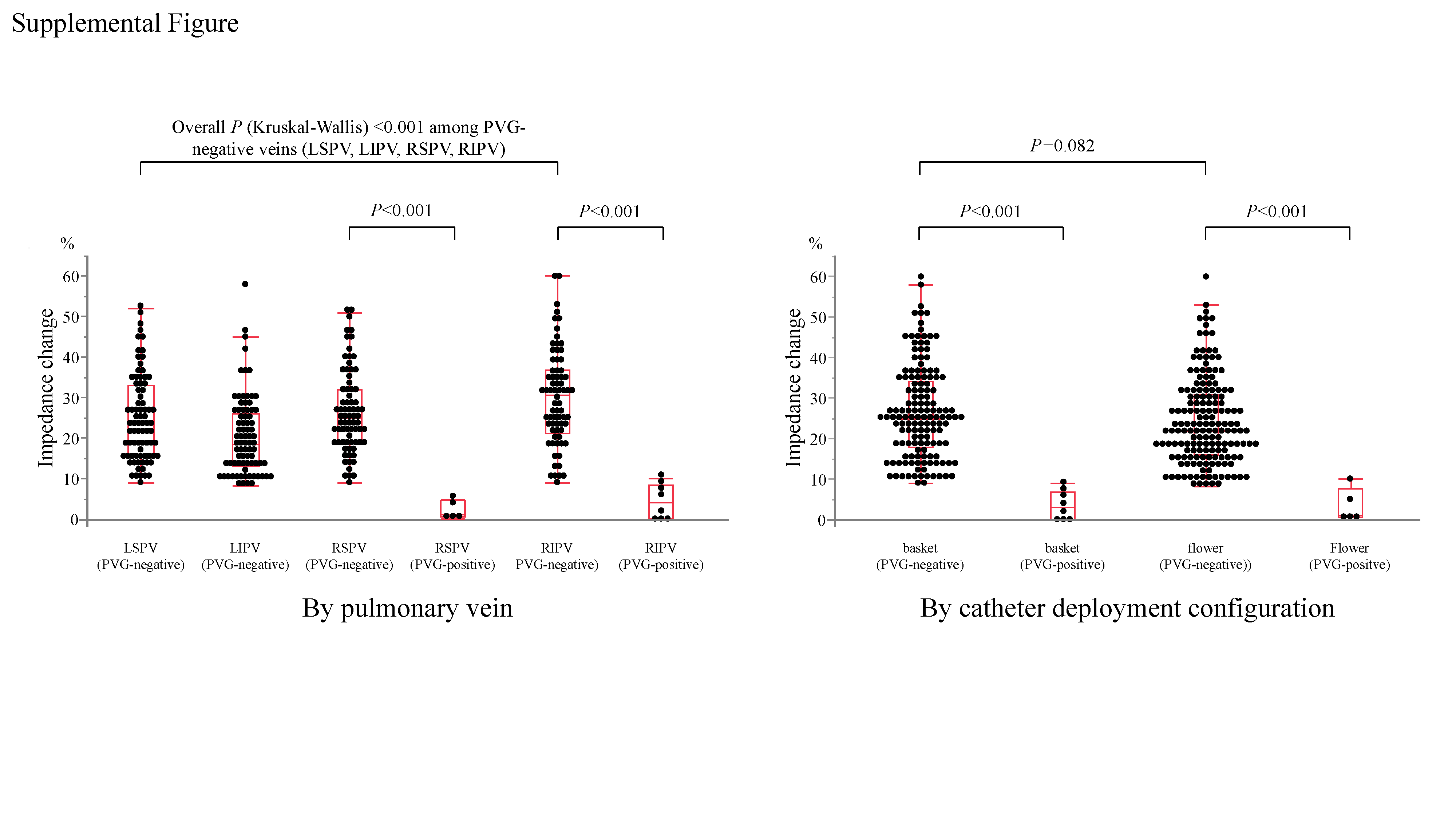

Supplement: Supplementary file 1 — Data S1: Figure S1. Distribution of impedance change (%) stratified by pulmonary vein gap (PVG) status. Left panel: By pulmonary vein—left superior (LSPV), left inferior (LIPV), right superior (RSPV), and right inferior (RIPV)—shown separately for PVG‐negative and PVG‐positive segments. Right panel: By FARAPULSE catheter deployment configuration (basket vs. flower), again split by PVG status. Overall, PVG‐positive segments show markedly lower impedance change than PVG‐negative segments across veins and configurations. [file JOA3-42-e70333-s001.tif]
